# The Wound–Heart Axis: Can Chronic Wounds Contribute to Cardiac Dysfunction?

**DOI:** 10.3390/ijms27188138

**Published:** 2026-09-12

**Authors:** Preeti K. Chaudhary, Howard H. Chen, Lakshmi Pulakat

**Affiliations:** 1 Molecular Cardiology Research Institute, Tufts Medical Center, Boston, MA 02111, USA; preeti.chaudhary@tuftsmedicalcenter.org (P.K.C.); howard.chen@tuftsmedicalcenter.org (H.H.C.); 2 Department of Medicine, Tufts University, Boston, MA 02111, USA

**Keywords:** chronic wounds, inflammation, wound–heart axis, cardiovascular risk, cardiac dysfunction

## Abstract

Traditionally, chronic wounds including diabetic foot ulcers (DFUs), pressure ulcers (PUs), venous leg ulcers (VLUs), arterial ulcers, inflammatory and autoimmune-associated ulcers, infected chronic wounds, and non-healing post-surgical wounds are treated as localized diseases of skin and soft tissue damage. However, these lesions are increasingly acknowledged as chronic inflammatory states with consequences extending beyond the wound bed and often arising in the setting of systemic dysfunction. Across wound types, shared features include persistent inflammation, oxidative stress, endothelial dysfunction, immune imbalance, protease dysregulation, infection or biofilm burden, metabolic disturbance, and impaired regenerative signaling. These abnormalities may promote systemic cytokine release, vascular dysfunction, neurohumoral activation, oxidative injury, and maladaptive remodeling. Cardiac dysfunction is already known to impair wound healing. In contrast, whether chronic non-healing wounds are associated with or may contribute to cardiac damage resulting in heart failure remains insufficiently defined. Emerging epidemiologic and mechanistic evidence suggests that chronic non-healing wounds may amplify cardiovascular stress, particularly in vulnerable patients with diabetes, obesity, frailty, kidney disease, or pre-existing vascular disease. This narrative review explores the conceptual hypothesis that chronic non-healing wounds may contribute to systemic cardiovascular stress and cardiac dysfunction. It integrates clinical and mechanistic opportunities, outlines potential pathways, including extracellular vesicle (EV) signaling, and highlights key knowledge gaps and therapeutic implications in the wound–heart axis.

## 1. Introduction

Chronic wounds are defined as wounds that fail to progress through an orderly and timely healing response via the normal phases of repair and do not achieve full anatomical and functional restoration. They include diabetic foot ulcers (DFUs), venous leg ulcers (VLUs), arterial and mixed ischemic ulcers, inflammatory and autoimmune-associated ulcers, infected chronic wounds, non-healing post-surgical wounds, and bed sores and crush injuries that do not heal and progress to chronic pressure ulcers (PUs). Although often approached as local defects of skin and soft tissue, chronic wounds are increasingly understood as manifestations of systemic pathology characterized by persistent inflammation, microvascular dysfunction, metabolic disturbance, immune dysregulation, and impaired reparative signaling [1,2].

The clinical burden of chronic wounds is substantial and often described as a silent epidemic in developed countries, costing the United States Medicare an estimated $22.5 billion annually. The global cost of wound care is estimated to be a staggering $148.65 billion [3,4]. These wounds are associated with pain, infection, prolonged hospitalization, immobility, impaired quality of life, and high recurrence. They frequently arise in patients with diabetes, advanced age, chronic kidney disease, frailty, and cardiovascular disease (CVD).

The relationship between heart and wound healing is well established from the perspective of cardio-metabolic diseases in causing chronic wounds. Diabetes- and obesity-induced ischemic CVDs and onset of heart failure that cause reduced perfusion, impaired microcirculation and oxygen delivery, endothelial dysfunction, maladaptive neurohumoral activation, and systemic inflammation can all attenuate tissue repair, causing chronic wounds [5,6]. By contrast, whether chronic wounds reciprocally contribute to cardiac stress and dysfunction remains incompletely understood. This question is clinically relevant because chronic wounds are persistent sources of inflammatory, oxidative, metabolic, and hemodynamic stress and infections that can lead to fatal pathologies such as sepsis [7,8]. In principle, these signals could extend beyond the wound bed and influence distant organs, including the heart [1,9]. Even in otherwise healthy individuals without pre-existing CVD or major cardiometabolic risk factors, a persistent non-healing wound may become a sustained source of systemic inflammatory stress capable of causing cardiac dysfunction and damage [10,11,12]. In patient populations with obesity, diabetes, advanced age, or frailty, who may have their cardiovascular reserve already compromised, the cardiovascular risk may be exacerbated, and myocardium more susceptible to structural and functional damage from the increased inflammatory insult arising from chronic wounds.

A deeper understanding of the molecular continuum linking chronic wound biology to myocardial injury is essential. The primary aim of this narrative review is to explore and develop the emerging hypothesis that chronic non-healing wounds may act as active amplifiers of systemic cardiovascular stress and potentially contribute to cardiac dysfunction. A secondary aim is to qualitatively map and integrate the available clinical, epidemiological, and mechanistic evidence relevant to this proposed wound–heart axis. Thus, in this narrative review with a conceptual focus, we first summarize the clinical burden and cardiovascular relevance of major chronic wound phenotypes. We then outline plausible mechanisms linking chronic wounds to cardiac dysfunction, including endothelial injury, cytokine-mediated stress on myocardium, profibrotic remodeling, oxidative and mitochondrial stress, maladaptive neurohumoral activation, infection-related innate immune signaling, anemia caused by chronic inflammation, and physical deconditioning. We also discuss extracellular vesicle (EV)-mediated signaling as a candidate mechanism for wound-to-heart communication and identify major knowledge gaps in this field and future therapeutic directions.

### Review Approach and Literature Search

This article was developed as a narrative review with a conceptual focus. The literature search was selective and narrative, although intentionally broad in disciplinary scope. We have included the representative evidence from wound biology, cardiovascular medicine, immunology, and vascular biology to evaluate the biological plausibility of a wound–heart axis. Relevant literature was identified through searches of PubMed, Google Scholar, and Scopus up to 24 June 2026. Search terms included combinations of “chronic wound,” “pressure ulcer,” “diabetic foot ulcer,” “venous leg ulcer,” “arterial ulcer,” “non-healing surgical wound,” “crush injury,” “cardiac dysfunction,” “heart failure,” “cardiovascular disease,” “systemic inflammation,” “endothelial dysfunction,” “extracellular vesicles,” and “microRNA.” Reference lists of relevant articles and reviews were also examined to identify additional publications. Peer-reviewed clinical, epidemiological, experimental, and mechanistic studies, together with reviews, meta-analyses, and relevant clinical guidelines, were selected based on their relevance to chronic wound biology, systemic consequences of wounds, cardiovascular outcomes, and potential wound-to-heart signaling mechanisms. Exclusion criteria included duplicate publications; studies unrelated to chronic or non-healing wounds; studies lacking sufficient methodological or outcome information; non-peer-reviewed publications and articles not published in English. The review was not intended to provide an exhaustive or systematic survey of all studies in these fields.

## 2. Major Chronic Wound Phenotypes and Their Cardiovascular Relevance

**Figure 1:**
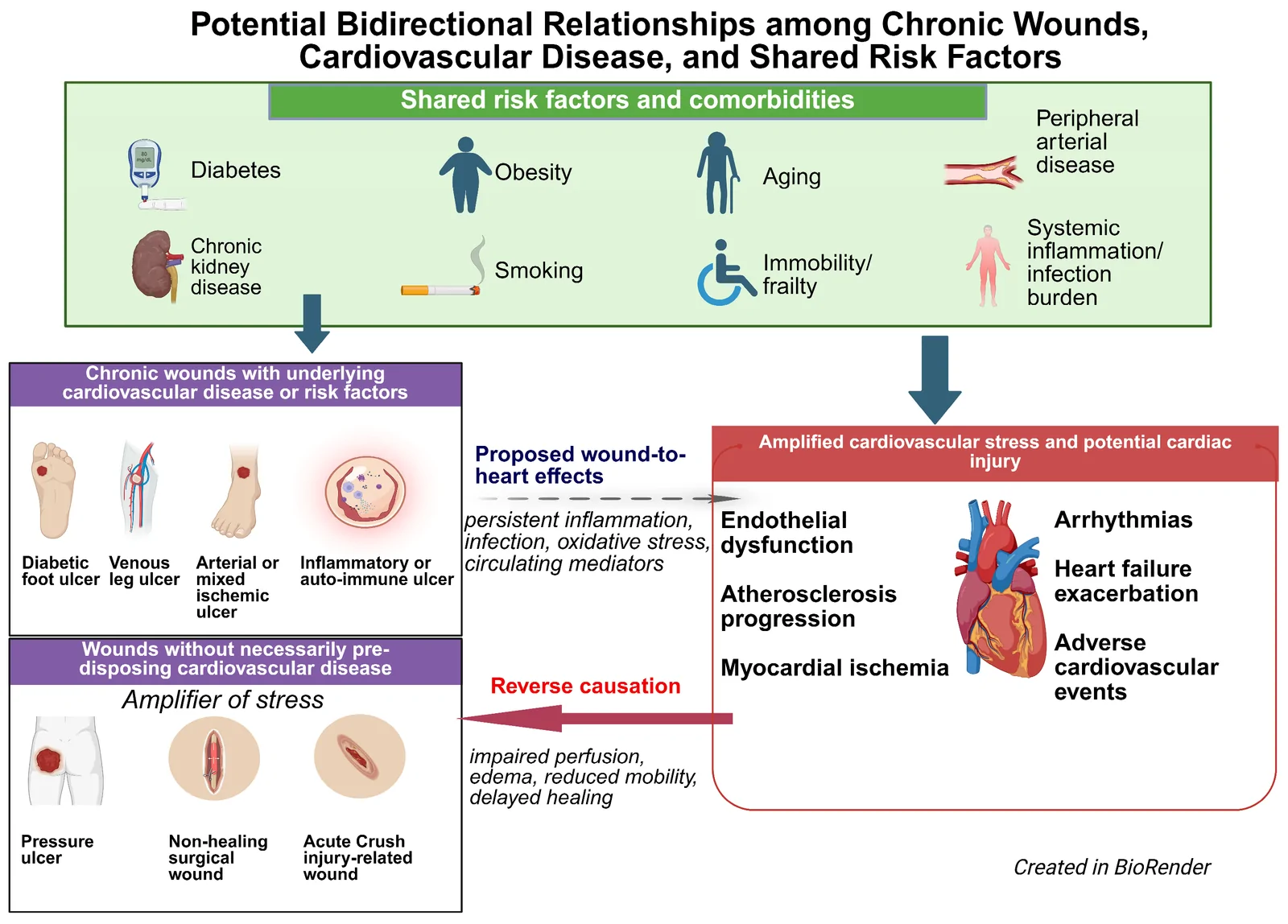
Proposed wound–heart axis across chronic wound phenotypes. Chronic wounds may arise either in the setting of underlying cardiovascular disease (CVD) or cardiometabolic risk factors (e.g., diabetic foot, venous, arterial/mixed ischemic, and inflammatory ulcers) or in contexts not necessarily defined by predisposing CVD (e.g., pressure ulcers, non-healing surgical wounds, and chronic ulcers caused by crush injury). Regardless of origin, once established, these wounds may be associated with systemic cardiovascular stress. Their potential contribution to cardiac injury, including endothelial dysfunction, atherosclerosis progression, myocardial ischemia, arrhythmia, heart failure exacerbation, and adverse cardiovascular events may vary according to wound type, infection burden, comorbidities, and underlying cardio-metabolic and immune diseases. Created in BioRender. Chaudhary, P. (2026) https://BioRender.com/5rs4ieu.

Chronic ulcers comprise a heterogeneous group of wounds with diverse etiologies. First, DFUs, VLUs, arterial and mixed ischemic ulcers, and inflammatory or autoimmune-associated ulcers develop as chronic ulcers due to underlying crush injury, diabetes, peripheral vascular disease, venous hypertension, and autoimmune diseases that independently increase cardiovascular risk (Table 1 and Table 2). In these settings, the chronic wound primarily serves as a marker of pre-existing cardiovascular vulnerability [13]. Second, once established, any chronic wound may act as a mediator or amplifier of systemic stress as it may become an independent source of persistent inflammation, oxidative stress, infection or biofilm burden, pain, anemia, immobility, and increased metabolic demand that, in turn, exacerbate cardiac stress [7,14,15]. This role may be particularly relevant to advanced PUs, infected DFUs, recurrent venous ulcers, and non-healing post-surgical or crush-injury-induced chronic ulcers. Third, bed sores and non-healing wounds caused by surgery or crush injury may arise in patients without prior diagnosis of CVD. Such wounds could potentially contribute to new-onset CVD as they serve as a persistent source of systemic inflammatory markers that can affect cardiovascular health. Such chronic wounds exemplify clinically relevant settings for investigating whether a chronic wound represents a potential independent contributor to cardiovascular disease (Figure 1). Accordingly, chronic wounds can not only be consequences of underlying cardiometabolic or autoimmune diseases, but also potential amplifiers of cardiovascular stress (Figure 1).

### 2.1. Pressure Ulcers (Ischemic-Immobility-Associated Wounds)

PUs arise from prolonged mechanical loading, usually exerted over bony prominences, that compromises local perfusion, resulting in ischemic tissue injury, hypoxia, and necrosis. They are most frequently seen in elderly populations, critically ill patients, immobile patients, and people with spinal cord injuries. In the United States, approximately 2.5 million PUs are reported in acute-care facilities, although this estimate does not include individuals living at home or in nursing facilities [16]. Pressure-injury incidence can vary across settings. Assessment of prospective cohorts from four intensive care units (ICUs) in a Norwegian university hospital reported a 12-month cumulative incidence of 29%, or 16% when category I injuries were excluded [17]. A retrospective study from ICUs affiliated with Ardabil University of Medical Sciences in Iran reported a three-year cumulative incidence of 13.56% [18]. In addition to causing local tissue damage, PUs are closely linked to higher mortality, longer hospital stays, a higher risk of infection, and a significant financial burden [3]. Advanced stage III/IV ulcers are frequently complicated by deep infection, osteomyelitis, anemia, and recurrent bacteremia [19]. Current guidelines also recommend performing blood cultures when systemic infection is suspected and bone biopsy when osteomyelitis is suspected [20]. In addition to the above-described systemic disease burden, Stage IV PUs may contribute to cardiac damage; however, this possibility has not been well defined clinically. Accordingly, no current study or guideline supports routine screening for cardiac damage or heart failure solely on the basis of a Stage IV PU. Currently, management is focused on pressure relief, nutritional support, infection control, debridement, and reconstruction when appropriate. There are no effective treatments that support full recovery of advanced PUs, such as Stage IV PUs that are reported to have a 25–40% increase in mortality risk within one year compared to patients without ulcers. Whether the Stage IV PU-induced high mortality risk is caused, in part, by the persistent inflammation and cardiovascular risk arising from the ulcer itself (in addition to underlying diseases) is not explored in the literature.

### 2.2. Diabetic Foot Ulcers (Metabolic-Neurovascular Wounds)

DFUs affect approximately 19–34% of people with diabetes at some point in their lives [21]. These ulcers develop through the combined effects of peripheral neuropathy, impaired microvascular perfusion, hyperglycemia-induced immune dysfunction, and repetitive mechanical stress [21]. Chronic hyperglycemia promotes advanced glycation end product formation, oxidative stress, endothelial dysfunction, and defective macrophage polarization, all of which impair wound resolution [2]. Beyond their local pathological effects, DFUs are increasingly recognized as markers of heightened systemic cardiovascular risk. Recent evidence indicates that, compared with individuals with diabetes without DFU, patients with DFU have significantly higher risks of ischemic heart disease, stroke, and cardiovascular mortality [13]. A 2024 narrative review further proposed a ‘cardio-renal-metabolic-foot’ connection, highlighting that patients with DFU often carry a greater burden of cardiovascular risk factors, heart failure, and chronic kidney disease, with more than a two-fold higher risk of cardiovascular death; however, that review also emphasized that the clinical value of routine cardiovascular screening in DFU remains to be established [22]. Early supportive clinical data suggest that cardiovascular assessment may still be relevant in selected patients: electrocardiogram (ECG) and natriuretic peptide testing have been proposed when cardiac disease is suspected. Echocardiography may aid earlier detection of subclinical dysfunction, although these approaches have not yet been incorporated into formal DFU screening guidelines [22]. Consistent with this, an observational study from England found that incorporating a 12-lead ECG into routine multidisciplinary DFU care was feasible and frequently prompted medication adjustments or cardiology referral. However, this study did not demonstrate a statistically significant reduction in overall mortality from incorporating this approach [23].

### 2.3. Venous Leg Ulcers (Inflammatory-Venous Hypertensive Wounds)

The most prevalent kind of chronic wound, VLUs affect approximately 1% of the general population and account for over 70% of all leg ulcers [24]. These wounds result from persistent venous hypertension, microvascular congestion, and tissue hypoxia caused by anatomical or functional defects of venous valves [25]. The resulting hemodynamic disturbances promote leukocyte trapping, endothelial activation, capillary leakage, and persistent local inflammation that ultimately disrupt skin barrier integrity and impair wound healing [25,26]. The development of venous ulcers is caused by a number of risk factors, including advanced age, a family history of venous disease, hypertension, physical inactivity, chronic obstructive pulmonary disease (COPD), and previous deep vein thrombosis (DVT) [27]. VLUs occur within the spectrum of advanced chronic venous disease that contribute to excess cardiovascular morbidity and mortality. A recent systematic review and meta-analysis reported that patients with advanced chronic venous disease, particularly those with clinical-etiological-anatomical-pathophysiological classification (CEAP) C5 (healed venous ulcer) and C6 (active venous ulcer), have increased cardiovascular events and cardiovascular death [28].

### 2.4. Arterial and Mixed Ischemic Ulcers

Arterial insufficiency ulcers develop in the context of peripheral artery disease (PAD) and reduced macrovascular perfusion [29]. These wounds reflect advanced systemic atherosclerosis and are often accompanied by coronary artery disease and cerebrovascular disease [29,30]. Mixed arterial-venous ulcers further illustrate the overlap between systemic vascular pathology and chronic wound formation. In such cases, concomitant PAD may underlie since nonhealing ulcers of mixed etiology may benefit from arterial revascularization [31]. Arterial ulcers, particularly those occurring in chronic limb-threatening ischemia, are manifestations of advanced PAD and identify a population at very high cardiovascular risk, with increased risks of myocardial infarction, stroke, and death [30]. Recent data show that patients with chronic limb-threatening ischemia are at substantial risk for major adverse cardiovascular events (MACE). Subsequently, 1-year MACE prediction models have been developed for this population [30]. However, the extent to which the presence, severity, or persistence of arterial ulcers independently contributes to cardiovascular events remains unclear.

### 2.5. Inflammatory and Autoimmune-Associated Ulcers

Certain chronic wounds arise primarily from immune dysregulation rather than perfusion deficits. Examples include pyoderma gangrenosum, vasculitis ulcers, and autoimmune connective tissue disease-associated ulcers. These wounds are characterized by sterile inflammation, excessive neutrophil infiltration, and dysregulated cytokine production. Recent evidence indicates that pyoderma gangrenosum is independently associated with major adverse cardiovascular events [32]. Anti-neutrophil cytoplasmic antibody (ANCA)-associated vasculitis is associated with increased risks of ischemic heart disease, myocardial infarction, stroke, heart failure, and cardiovascular mortality [33]. Similarly, cutaneous lupus erythematosus is associated with increased atherosclerotic cardiovascular disease risk [34]. Systemic sclerosis guidelines now recommend structured longitudinal cardiopulmonary and cardiovascular surveillance, including annual screening with echocardiography, cardiac damage biomarkers, and electrocardiography in selected contexts [35]. However, current evidence supports cardiovascular assessment primarily on the basis of the underlying autoimmune or vasculitic disorder rather than the ulcer itself. Thus, inflammatory and autoimmune-associated ulcers can be regarded as clinical markers of possible systemic disease requiring broader cardiovascular risk evaluation.

### 2.6. Infectious Chronic Wounds

Persistent infection and biofilm formation are common features of many chronic wounds, particularly DFUs and advanced PUs [36]. Polymicrobial colonization sustains neutrophilic inflammation. This elevates circulating inflammatory mediators that are capable of adversely affecting cardiac function [37].

### 2.7. Non-Healing Surgical and Post-Operative Wounds

Surgical wounds that fail to heal, particularly in obese, diabetic, or cardiac patients, represent another important category of chronic wounds. Impaired perfusion, systemic inflammation, renin–angiotensin–aldosterone system (RAAS) activation, and oxidative stress may contribute to delayed healing in this population. Post-operative wound complications are associated with prolonged hospitalization. These patients may also have increased cardiovascular vulnerability, especially in high-risk surgical cohorts with underlying cardiovascular comorbidities [38]. In these patients, myocardial injury after noncardiac surgery (MINS) and/or severe infection and subsequent sepsis-induced myocardial dysfunction can occur. These events represent plausible mechanisms linking impaired wound recovery to cardiac dysfunction [38]. Accordingly, perioperative cardiovascular assessment, including postoperative troponin surveillance in selected high-risk patients, is increasingly recommended for patients with known CVD, cardiovascular risk factors, or those undergoing elevated-risk surgery [38]. Although postoperative wound complications can potentially exacerbate cardiovascular risk, no current guideline recommends routine cardiac troponin or other cardiac biomarker surveillance solely because a surgical wound has failed to heal.

### 2.8. Crush Injury-Induced Chronic Ulcers

Crush injuries resulting from prolonged entrapment under crushing external pressure per se are not chronic wounds if they heal appropriately. Hazardous environments such as earthquake zones, war zones, structural collapse, or vehicle accidents often cause crush injuries. The immediate concern in patients with crush injuries is the development of Crush syndrome that can be fatal. While Crush Syndrome itself can cause significant cardiac risk as described below, here we are considering post-crush injury complications such as the development of chronic high-grade ulcers that occur later in victims of crush injury after decompression.

Crush Syndrome-related acute cardiac stress: Prolonged compression causes muscle ischemia, necrosis, and traumatic rhabdomyolysis. Once the external force is removed and compression is relieved, reperfusion leads to the systemic release of potassium, myoglobin, phosphate, organic acids, and inflammatory mediators [39]. At the same time, fluid may shift from the intravascular space into injured and edematous tissues, a process known as third-spacing, which can reduce circulating blood volume and worsen hypovolemia. Together, rhabdomyolysis-related electrolyte and acid-base disturbances, hemorrhage, and third-spacing increase the risk of hypovolemic shock, hyperkalemia-induced arrhythmias, cardiac arrest, and myocardial ischemia–reperfusion injury [40]. Importantly, early arrhythmogenic risk is driven largely by potassium and acid-base abnormalities from muscle injury, rather than blood loss alone. Experimental studies in rats have demonstrated persistent ECG abnormalities after crush injury and decompression, and later ventricular remodeling has also been described [40]. Current civilian, disaster-response, and military guidance therefore emphasize early trauma care, tailored fluid resuscitation, potassium assessment when available, and ECG-based monitoring for hyperkalemia-related dysrhythmia during entrapment, release, and evacuation [41,42]. However, survivors of crush injury and immediate acute life-threatening conditions such as Crush Syndrome often develop prolonged immobility, tissue ischemia, edema, and impaired perfusion that lead to secondary PUs. Thus, crush injuries often cause chronic wound development that, in turn, can amplify cardiac risk. However, no current guideline recommends routine screening for myocardial injury or heart failure solely on the basis of crush injury or prolonged entrapment. Management remains focused on hemodynamic stabilization, arrhythmia prevention, renal protection, and treatment of confirmed hyperkalemia. Importantly, direct evidence that cardiac stress can be increased due to a persistent or non-healing wound that may develop as a consequence of crush injury is currently lacking.

A summary of clinical evidence linking chronic wound-related pathology with cardiovascular outcomes is presented in Table 1 and Table 2. Although several studies demonstrate significant cardiovascular associations after multivariable adjustment, the current evidence does not conclusively establish an independent causal contribution of the wound itself.

**Table 1:** Wound-associated clinical and mechanistic evidence linking chronic wounds with cardiovascular outcomes.

Wound Type	Population/Comparison	Cardiovascular or Mortality Outcome	Effect Estimate (95% CI, Where Available)	Principal Covariates/Confounders	Proposed Mechanism	Supporting Literature
Active DFU	324 patients with diabetes: DFU (*n* = 162) vs. diabetes without foot ulcer (*n* = 162)	No direct cardiac outcome. DFU patients had higher circulating IL-6, hsCRP, and TNF-α and lower adiponectin independent of concomitant infections	Not reported for a cardiovascular outcome.	Concomitant infection was evaluated; matched for age, sex, and body mass index; associations with ulcer grade, lipids, retinopathy, nephropathy, hypertension, smoking, and other clinical variables were examined	Persistent wound-associated inflammatory activation and reduced cardioprotective adiponectin may contribute to systemic cardiovascular stress	Zubair et al. [43]
DFU	Meta-analysis of 10 studies involving >8000 patients; diabetes with DFU vs. diabetes without DFU	Ischemic heart disease, cerebrovascular events/stroke, cardiovascular-related mortality; heart failure also evaluated	IHD: RR 1.25; CVA: RR 2.03; CHF: RR 1.63; cardiovascular-related mortality: RR 2.59	No uniform covariate set across studies. Included observational studies differed in adjustment for cardiovascular and diabetes-related comorbidities	DFU-associated inflammation, infection, endothelial dysfunction, and vascular stress may contribute to cardiovascular risk	Chin et al. [13]
Active DFU stratified by depth and infection	Prospective cohort of 513 patients with DFU; deep + infected vs. non-deep/non-infected ulcers	MACE: hospitalization for MI, stroke/TIA, or HF; also MACE + all-cause mortality	MACE: adjusted HR) 3.61 (1.59–8.17); MACE or mortality: adjusted HR 3.03 (1.62–5.67) for deep + infected vs. non-deep/non-infected DFU	Age, social deprivation, diabetes type, hypertension, dyslipidemia, smoking, SGLT2i/GLP-1RA use, atherosclerotic CVD including CHD/PAD/stroke, HF, nephropathy, other comorbidities, wound area, and wound location	Greater tissue injury and infection may amplify systemic inflammation and thereby contribute to increased cardiovascular risk	Lan et al. [44]
PG, an ulcerative neutrophilic dermatosis	227 PG cases and 882 matched controls; cases were matched 4:1 with controls by age, sex, ethnicity, BMI, and smoking status in the US	MACE, including MI, cerebrovascular accident, HF, acute coronary syndrome, unstable angina, ischemic heart disease, cardiac arrest, and angina	Adjusted OR 2.19 (95% CI 1.47–3.27)	Hypertension, diabetes mellitus, hyperlipidemia, SLE, and rheumatoid arthritis; matching included age, sex, ethnicity, and smoking	Neutrophilic and systemic inflammatory activation associated with PG may promote vascular inflammation and cardiovascular injury	Adjei-Frimpong et al. [32]

Note: IHD: Ischemic heart disease; RR: risk ratio; CVA: Cerebrovascular accident; CHF: Congestive heart failure; MACE: Major adverse cardiovascular events; HR: Hazard ratio; SGLT2i: Sodium-glucose cotransporter-2 inhibitors; GLP-1RA: Glucagon-like peptide-1 receptor agonists; CVD: Cardiovascular disease; PG: Pyoderma gangrenosum; MI: Myocardial infarction; CI: Confidence interval; SLE: Systemic lupus erythematosus.

**Table 2:** Underlying disease and confounding evidence relevant to interpretation of the wound–heart axis.

Wound Type	Population/Comparison	Cardiovascular or Mortality Outcome	Effect Estimate (95% CI, Where Available)	Principal Covariates/Confounders	Proposed Mechanism	Supporting Literature
DFD including ulceration and more severe foot complications	11,117 T2D patients with first DFD episode vs. 1:1 propensity-score-matched T2D patients without DFD	Cardiovascular events and all-cause mortality	CV events: HR 2.27 (2.17–2.38); mortality: HR 2.24 (2.15–2.33)	Propensity matching: age, sex, inclusion year, diabetes duration, hypertension, hyperlipidemia, retinopathy, neuropathy, CKD, PAD, IHD, stroke, and HF. Adjusted Cox models additionally considered smoking, alcohol consumption, BMI, and HbA1c.	Severe DFD may reflect cumulative systemic microvascular and macrovascular disease, with a possible additional inflammatory burden.	Vlacho et al. [45]
At-risk diabetic foot without chronic ulcer, defined by PAD and/or peripheral neuropathy	946 people with diabetes; at-risk foot (*n* = 301) vs. no at-risk foot (*n* = 645), NHANES	Cardiovascular mortality and all-cause mortality	CV mortality: HR 2.494 (1.809–3.438); all-cause mortality: HR 2.050 (1.524–2.758)	Age, sex, race, BMI, smoking, total cholesterol, ALT, AST, hypertension, CKD, and pre-existing cardiovascular disease	PAD, neuropathy, and systemic vascular disease themselves confer substantial cardiovascular risk	Wu et al. [46]
CVD, including advanced CEAP stages; C5 = healed venous ulcer and C6 = active venous ulcer	Systematic review of 20 observational studies; meta-analysis of 6 studies involving 393,875 individuals and 55,356 cardiovascular events	Coronary artery disease, stroke, PAD, HF, cardiovascular mortality, and other cardiovascular outcomes	Associations across included studies generally ranged from OR/HR 1.3–3.8; quantitative analysis reported an adjusted expected OR 2.50 (95% CI 1.15–5.44); heterogeneity was high	Covariate adjustment varied across studies; population-based studies adjusted for major cardiovascular risk factors including age, sex, hypertension, diabetes, obesity, dyslipidemia, and smoking	Systemic inflammation, endothelial dysfunction, venous hypertension, and broader vascular dysfunction may link CVD with cardiovascular disease	Del Rio-Sola et al. [28]
CLTI, which may include ischemic nonhealing ulceration and/or gangrene	1780 patients with CLTI from the BEST-CLI trial; model externally validated in the BEST Registry	1-year MACE (composite of all-cause death, MI, and stroke)	1-year MACE incidence: 17.33% (95% CI 15.59–19.24%). Prior CAD: HR 2.22 (1.72–2.89); HF: HR 1.93 (1.35–2.75); CKD stage ≥3: HR 1.46 (1.14–1.87)	Multivariable risk modeling incorporated demographic and clinical cardiovascular risk factors; major predictors included age, BMI, hypertension, CAD, HF, CKD, diabetes, and statin use	Advanced systemic atherosclerosis and associated cardiac and renal comorbidities create a high cardiovascular-risk state	Vemulapalli et al. [47]

Note: DFD: Diabetic foot disease; T2D: Type 2 diabetes; CV: Cardiovascular; HR: Hazard ratio; CKD: Chronic kidney disease; PAD: Peripheral artery disease; IHD: Ischemic heart disease; HF: Heart failure; BMI: Body mass index; NHANES: National Health and Nutrition Examination Survey; CEAP: Clinical-Etiological-Anatomical-Pathophysiological classification; CVD: Chronic venous disease; OR: Odds ratio; HR: Hazard ratio; CLTI: Chronic limb-threatening ischemia; BEST-CLI: Best endovascular versus best surgical therapy for patients with critical limb ischemia; MACE: Major adverse cardiovascular events; MI: Myocardial infarction; CI: Confidence interval; CAD = Coronary artery disease; CKD = Chronic kidney disease.

## 3. Molecular and Cellular Mechanisms Linking Chronic Wounds to Cardiac Dysfunction

Chronic non-healing wounds are frequently accompanied by persistent local inflammatory and physiologic stress that may extend beyond the wound site. The myocardium is particularly vulnerable to sustained exposure to inflammatory mediators, oxidative stress, endothelial dysfunction, maladaptive neurohumoral activation, infection-related signaling, impaired oxygen delivery, and physical deconditioning. Although direct causal evidence in humans remains limited, several interrelated pathways support a credible mechanistic basis for a wound–heart axis.

### 3.1. Endothelial Axis: From Chronic Wound Inflammation to Cardiac Dysfunction

One of the earliest and most plausible links between chronic wounds and cardiac dysfunction is endothelial injury. Human studies show that chronic wounds have significant and clinically relevant systemic inflammatory effects. Chronic wounds cause an increase in inflammatory markers such as tumor necrosis factor-alpha (TNF-α), interleukin-1 beta (IL-1β), interleukin-6 (IL-6), and C-reactive protein (CRP) [10,11,43]. These molecules damage vascular endothelium, increase leukocyte adhesion, reduce nitric oxide bioavailability, and impair vasodilatory responsiveness. Endothelial dysfunction has direct cardiac consequences. Reduced nitric oxide signaling and oxidative stress impair coronary microvascular function, promote vascular stiffening, and contribute to abnormal ventricular-vascular coupling [48,49]. Over time, these changes may reduce myocardial oxygen delivery and are mechanistically linked to myocardial ischemia, diastolic dysfunction, and heart-failure progression [49]. Thus, chronic wound-driven endothelial dysfunction offers an example of a mechanistic bridge between peripheral tissue injury and cardiovascular dysfunction.

### 3.2. Cytokine-Myocardial Axis: Direct Effects of TNF-α and IL-6 on the Heart

A second major mechanism is the direct effect of circulating inflammatory cytokines on myocardial cells. Among these, TNF-α is especially important because it is a central mediator of chronic wound inflammation and a recognized modulator of cardiac function. Sustained TNF-α exposure has negative inotropic effects and disrupts excitation-contraction coupling by altering calcium-handling pathways, including phospholamban-dependent regulation of sarcoplasmic reticulum Ca^2+^-ATPase 2a (SERCA2a) [50]. These changes impair intracellular calcium reuptake, reduce contractile efficiency, and may promote cardiomyocyte apoptosis and adverse remodeling. In the setting of prolonged inflammatory exposure, such effects are biologically capable of contributing to progressive systolic dysfunction.

IL-6 provides an additional pathway linking chronic wounds to myocardial injury. Persistent IL-6 signaling activates gp130/Janus kinase (JAK)/signal transducer and activator of transcription 3 (STAT3)-associated pathways that promote inflammatory remodeling. This contributes to cardiac fibroblast activation, extracellular matrix accumulation, and myocardial stiffening [51,52]. Chronic IL-6 elevation has been associated with inflammatory heart-failure phenotypes, particularly heart failure with preserved ejection fraction (HFpEF), in which diastolic dysfunction, microvascular inflammation, and interstitial fibrosis are prominent features [50]. Together, this impact of TNF-α and IL-6 offers a mechanism that potentially defines how a chronic wound may function as a sustained peripheral source of cardiotoxic inflammatory signaling.

### 3.3. Profibrotic Remodeling Axis: Transforming Growth Factor-Beta (TGF-β) and Extracellular Matrix Accumulation

Persistent inflammation can converge on profibrotic signaling pathways, particularly those mediated by TGF-β, a cytokine family comprising three mammalian isoforms (TGF-β1, TGF-β2, and TGF-β3). Among these, TGF-β1 is the principal isoform implicated in inflammation-driven tissue injury, fibrosis, and adverse cardiac remodeling [53,54]. Chronic activation of TGF-β1 signaling promotes fibroblast-to-myofibroblast transition, collagen deposition, extracellular matrix expansion, and increased myocardial stiffness [54,55,56,57]. These changes impair ventricular relaxation, reduce compliance, and progressively favor diastolic dysfunction [58]. This mechanism is particularly compelling because chronic wounds themselves are characterized by dysregulated matrix turnover, persistent protease activity, impaired matrix deposition, and prolonged inflammatory signaling. If this inflammatory state is sustained systemically, it is biologically feasible that it may also drive maladaptive matrix remodeling within the heart. Thus, both structural remodeling and functional myocardial depression may be influenced by chronic wounds.

### 3.4. Oxidative and Mitochondrial Axis: Energetic Stress in the Myocardium

Excessive production of reactive oxygen species (ROS), poor antioxidant buffering, and mitochondrial dysfunction are linked to chronic wounds. Persistent wounds may potentially contribute to a wider systemic oxidative and inflammatory milieu, since they can serve as sources for elevated ROS levels and other molecules that increase oxidative stress and mitochondrial dysfunction in blood [59]. Cardiomyocytes are especially susceptible to these damaging molecules in the circulation because of their high mitochondrial density and continuous adenosine triphosphate (ATP) demand [60]. Systemic oxidative stress can cause lipid peroxidation, oxidative phosphorylation impairment, mitochondrial deoxyribonucleic acid (DNA) damage, and cardiomyocyte death. Through mitochondrial dysfunction, poor calcium handling, and unfavorable remodeling, even a slight but persistent redox imbalance can lower contractile reserve and compromise both systolic and diastolic performance. Thus, chronic wound-associated oxidative stress may act as a second hit that exacerbates cardiac failure in people with cardio-metabolic diseases and/or aging-related mitochondrial decline.

### 3.5. Neurohumoral Axis: Maladaptive Sympathetic Activation and RAAS Overactivation

Central stress circuits may be triggered by persistent nociceptive and inflammatory input from chronic wounds, especially painful or infected ulcers [15]. Persistent sympathetic nervous system activation increases heart rate, raises myocardial oxygen demand, promotes arrhythmogenicity, and causes overactivation of RAAS. Increased RAAS activity promotes sodium and water retention, expands intravascular volume, elevates preload and afterload, and thereby increases cardiac wall stress [61]. This maladaptive neurohumoral response results in vasoconstriction, sodium retention, oxidative stress, and adverse ventricular remodeling. Concurrently, angiotensin II-dependent TGF-β signaling, fibrosis, endothelial dysfunction, and inflammatory amplification are all promoted by RAAS activation. Thus, a neurohumoral environment similar to that observed in the early stages of heart failure progression may be influenced by systemic stress and persistent wound pain.

### 3.6. Infection–Endotoxin Axis: Innate Immune Activation Beyond the Wound Bed

The extent of microbial invasion and the host response may contribute to wound-associated cardiovascular implications. Colonization by microorganisms without tissue invasion or clinical infection, by itself, may not cause systemic cardiac injury. However, biofilm formation complicates the treatment of chronic infections by multidrug-resistant bacteria and can sustain local immune activation, delay wound resolution, and contribute to prolonged low-grade inflammatory signaling [36,62]. Intermittent bacterial translocation or low-grade endotoxin exposure may further maintain systemic immune activation even in the absence of overt sepsis [63]. These microbial signals can activate toll-like receptor (TLR) pathways, particularly TLR4, increase cytokine release, promote vascular inflammation, and potentially contribute to atherosclerotic plaque destabilization [64]. This mechanism impacts cardiac health. In these settings, repeated exposure of the myocardium and vasculature to inflammatory insults may increase vulnerability to decompensation, plaque instability, and progressive myocardial damage.

### 3.7. Hematologic–Metabolic Axis: Anemia, Chronic Inflammation and Oxygen Supply Mismatch

Chronic inflammation can suppress erythropoiesis through hepcidin-mediated iron sequestration and altered iron handling [65,66]. The resulting anemia from chronic inflammation reduces blood oxygen-carrying capacity and may increase cardiac workload through compensatory increases in cardiac output required to maintain tissue oxygen delivery [67]. Although tachycardia, greater myocardial oxygen demand, and increased cardiac output may initially aid in maintaining tissue perfusion, these adaptations may eventually turn maladaptive. In patients with limited cardiac reserve, chronic anemia may accelerate ventricular remodeling, worsen myocardial oxygen supply-demand mismatch, and hasten progression toward heart failure. This pathway is especially relevant in severe, longstanding ulcers, where ongoing inflammation, wound bioburden, and recurrent infection may sustain systemic inflammatory and hematologic stress over prolonged periods.

### 3.8. Deconditioning Axis: Immobility, Hemodynamic Deterioration, and Cardiac Reserve Loss

Reduced mobility is a hallmark of many severe chronic wounds, including advanced PUs, DFUs, venous ulcers, and non-healing post-surgical wounds. Long-term inactivity promotes venous stasis, decreases endothelial shear stress, weakens the skeletal muscle pump, and raises the risk of thrombosis. Physical deconditioning also reduces stroke volume, exercise tolerance, and cardiovascular reserve [68]. This is important because both prolonged inflammatory signals and declining hemodynamic fitness may then pose a challenge to the heart. Thus, immobility may exacerbate cardiovascular vulnerability in concert with oxidative stress, cytokine exposure, and neurohumoral activation.

Taken together, these pathways provide evidence for a credible mechanistic model in which chronic wounds are not merely markers of systemic illness but may also function as active amplifiers of cardiac stress.

## 4. Extracellular Vesicle-Associated miRNAs and Proteins from Chronic Wounds with Potential Cardiac Effects

Wound tissues and wound-derived EVs have been shown to contain a variety of regulatory microRNAs (miRNAs), inflammatory mediators, matrix-remodeling enzymes, and structural proteins [69,70,71]. Many of these molecules are known to participate in key biological processes involved in cardiovascular injury, including endothelial dysfunction, myocardial fibrosis, inflammatory signaling, oxidative stress, extracellular matrix remodeling, and cardiomyocyte apoptosis (Table 3 and Table 4). For example, miRNAs such as miR-21, miR-126, miR-29, miR-125b, and miR-200 family members are widely reported in wound healing environments while also playing established roles in myocardial remodeling, ischemia–reperfusion injury, cardiac fibrosis, and heart failure (Table 3 and Table 4). Similarly, proteins identified within wound-derived EVs, including matrix metalloproteinases (MMP1, MMP2, MMP9), myeloperoxidase (MPO), S100 family proteins, and cathepsin S are strongly implicated in vascular inflammation, extracellular matrix degradation, plaque instability, and adverse ventricular remodeling (Table 3 and Table 4).

**Figure 2:**
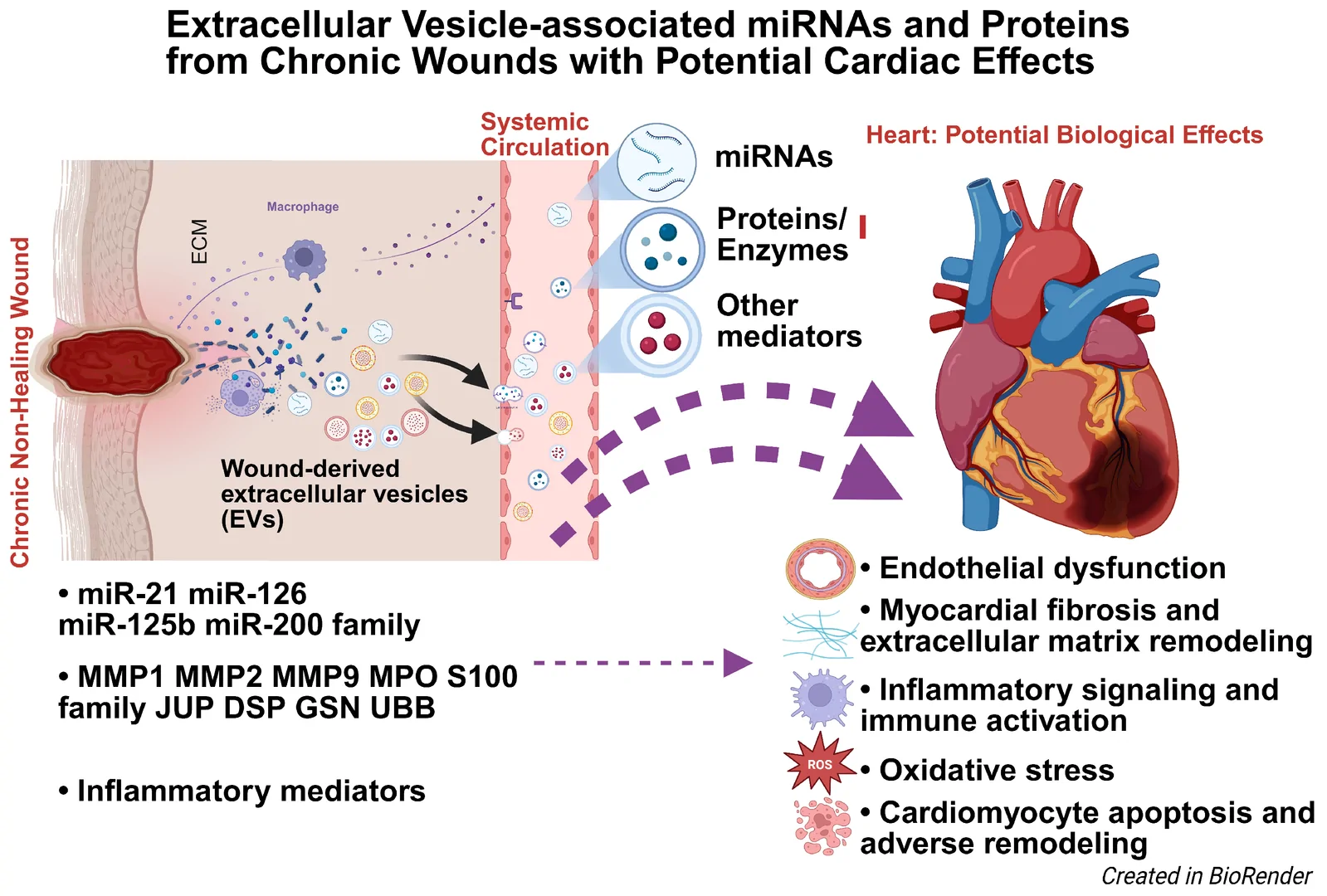
Extracellular vesicle (EV)-associated miRNAs and proteins from chronic wounds with potential cardiac effects. Chronic non-healing wounds may release EVs containing miRNAs, inflammatory mediators, matrix-remodeling enzymes, and structural or signaling proteins into the systemic circulation. These biologically active cargos may influence distant organs, including the heart, where they are implicated in endothelial dysfunction, vascular inflammation, immune activation, oxidative stress, myocardial fibrosis, cardiomyocyte apoptosis, and adverse remodeling. The dotted arrows indicate that transport of wound-derived EVs through the systemic circulation and their subsequent effects on the heart remain hypothetical and have not been directly demonstrated. Created in BioRender. Chaudhary, P. (2026) https://BioRender.com/ck1uvaw. ECM: Extracellular matrix; MMP: Matrix metalloprotease; MPO: Myeloperoxidase; JUP: Junction plakoglobin; DSP: Desmoplakin; GSN: Gelsolin; UBB: Ubiquitin.

These observations indicate that chronic wounds may release biologically active molecular cargo capable of influencing distant organs, including the heart (Figure 2).

The following tables (Table 3 and Table 4) summarize representative miRNAs and proteins identified in wound tissues or wound-derived EVs [69,70,71] and list their potential roles in cardiovascular injury or cardiac remodeling based on available data in the literature. Together, these data provide mechanistic support for a wound–heart signaling axis linking chronic non-healing wounds with cardiac dysfunction.

At present, however, the evidence remains largely mechanistic and hypothesis-generating. It is not yet known whether chronic wounds produce a distinct circulating vesicle signature that is directly cardiotoxic and how ulcer healing impacts such molecular signatures or their influence on cardiovascular health. For example, the elegant work by Soto et al., has defined unique protein-molecular signatures that distinguish DFU from VLU and that define their healing outcomes [69]. We examined the potential cardiovascular impact of these new DFU- and VLU-originated proteins (Table 4) and observed interesting associations. However, additional analysis is warranted to validate this connection. These questions remain central to defining whether EVs are biomarkers, mediators, or both that define the impact of ulcer progression or its mitigation on cardiovascular health status.

## 5. Knowledge Gaps and Future Research Directions

There are still significant knowledge gaps despite mounting epidemiologic and mechanistic data in favor of a potential wound–heart axis. The key unresolved issue is not simply whether chronic wounds coexist with CVD, but whether persistent non-healing ulcers independently contribute to cardiac stress, myocardial remodeling, and progression toward heart failure.

### 5.1. Does Successful Wound Healing Reduce Cardiovascular Risk?

Whether effective wound closure reduces cardiovascular risk is an important issue that warrants attention. Chronic ulcers are frequently seen as indicators of poor baseline health, diabetes, vascular disease, frailty, or immobility. However, chronic wounds may be contributors to cardiovascular damage as well as prognostic markers for assessing the impact of wound healing and associated reduction in systemic inflammatory burden on improving cardiac outcomes. It is still unclear if any improvement following healing is due to wound resolution per se or to associated beneficial modulation of confounding factors like baseline health, systemic inflammation, or care quality. This question is especially relevant in advanced PUs, infected diabetic foot ulcers, recurrent venous ulcers, and non-healing post-surgical or post-crush injury ulcers. Prospective studies that longitudinally track wound status and cardiovascular outcomes are needed to establish their temporal relationship. Mediation analyses incorporating inflammatory, endothelial, and cardiac biomarkers, including CRP, IL-6, TNF-α, troponin, and N-terminal pro-B-type natriuretic peptide (NT-proBNP), could further determine whether these pathways contribute to the association between chronic wounds and CVD.

### 5.2. Which Wound-Derived Inflammatory Signals Are Most Relevant to Cardiac Dysfunction?

Another major gap is the failure to distinguish general systemic inflammation from wound-derived signals that may be directly cardiotoxic. Chronic wounds differ in depth, ischemia, microbial burden, necrosis, and inflammatory composition. These differences may produce distinct systemic profiles with different cardiac consequences. Priority pathways include signaling mechanisms of TNF-α, IL-1β, IL-6, nuclear factor kappa-B (NF-κB), TGF-β, inflammasome activation, oxidative stress, endothelial injury, and cardiac stress biomarkers.

An important related question is whether specific wound classes map to specific cardiac pathologies. For example, severe immobility after crush injury may predispose to advanced PUs. PUs (whether from non-healing bed sores, post-crush injury ulcers, or post-surgical ulcers) may align more closely with immobility, infection, frailty, and deconditioning. DFUs align with increased expression and signaling by Advanced Glycation End Product-Receptor for advanced glycation end products (AGE-RAGE) signaling, metabolic stress, and microvascular dysfunction. Venous ulcers align with endothelial dysfunction and venous hypertension. Inflammatory and ischemic ulcers align with stronger cytokine-driven immune activation. Defining whether these wound-specific inflammatory patterns correspond to distinct cardiac outcomes, such as arrhythmias, myocardial dysfunction, ischemic events, heart failure decompensation, or adverse remodeling, remains an important area for future study.

### 5.3. Are Cardiac Effects Reversible After Chronic Wound Healing?

It is also unknown whether cardiac abnormalities associated with chronic wounds reverse after healing. If wound-associated cardiac effects are reversible, early wound closure becomes a systemic therapeutic goal rather than only a local endpoint. If they are only partially reversible, wound-directed care may need to be paired with cardioprotective strategies. Longitudinal studies using echocardiography, strain imaging, cardiac magnetic resonance imaging (MRI) where feasible, and serial biomarkers such as troponin, NT-proBNP, and fibrosis-associated markers would help define reversibility at structural, functional, and biochemical levels.

### 5.4. Do Extracellular Vesicles Mediate Wound-to-Heart Communication?

EVs represent one of the most compelling mechanistic candidates for wound–heart communication. Chronic wound tissue and wound-derived EVs have been shown to contain miRNAs, proteins, lipids, matrix-remodeling enzymes, and inflammatory cargo with known cardiovascular relevance (refer to Table 3 and Table 4). This may explain how a localized chronic ulcer can generate systemic effects even when circulating cytokine levels are only modestly elevated. A major unresolved question is whether chronic wounds produce a distinct EV signature that is directly cardiotoxic. Future work should isolate and compare circulating EVs from patients with chronic wounds, healed wounds, and matched controls without wounds.

### 5.5. Can Local Anti-Inflammatory Therapy Improve Both Wound Healing and Cardiac Outcomes?

Local anti-inflammatory therapy may improve tissue repair while lowering cardiac risk if wound-localized inflammation is a contributing factor to cardiovascular stress. This idea is particularly relevant for mediators like TNF-α, IL-1β, and IL-6. It is still unclear, though, which pathways can be safely targeted without compromising host defense or raising the danger of infection, and whether local drug treatments can reduce systemic inflammatory spillover without the drawbacks of systemic immunosuppression.

### 5.6. What Kind of Translational Model Is Needed to Prove the Wound–Heart Axis?

The absence of integrated models intended to investigate whether chronic wounds can cause heart dysfunction is impeding progress. Prolonged peripheral inflammatory injury is rarely included in cardiovascular models. The majority of wound models concentrate on local recovery, but longitudinal studies that assess cardiovascular stress are missing. Future models should include delayed wound closure, sustained inflammation, and, where relevant, bacterial burden or deep tissue injury, while also capturing cardiac structure, function, and biomarkers over time. It will be crucial to establish temporal orientation. Interventions that accelerate healing or suppress wound-localized inflammation should then test whether cardiac abnormalities improve in parallel with wound healing.

### 5.7. What Should Prospective Human Studies Measure?

Because they provide repeated measurement, temporal ordering, and clinical relevance, prospective human investigations are crucial. Research should simultaneously define the cardiovascular and wound phenotypes to advance the area. At the wound level, key variables include size, depth, duration, infection, recurrence, necrosis, osteomyelitis, and healing trajectory. At the systemic level, serial assessment should include inflammatory, endothelial, oxidative stress, and cardiac biomarkers, together with imaging-based measures of cardiac structure and function.

## 6. Current and Future Therapeutic Approaches for Chronic Wounds

Local treatment, which includes debridement, infection management, moisture-balanced dressings, offloading or pressure redistribution, revascularization where necessary, and negative pressure wound therapy, is still the mainstay of managing chronic wounds. The systemic inflammatory, metabolic, endothelial, and neurohumoral abnormalities that may accompany chronic non-healing wounds are not entirely addressed by these methods, despite their importance for wound bed preparation and tissue repair.

Medications including beta-blockers, angiotensin-converting enzyme (ACE) inhibitors or angiotensin receptor blockers, are widely used in the management of heart failure and selected cardiovascular conditions, whereas anti-lipidemic statins have been prescribed for the management of coexisting hypertension, and dyslipidemia [191,192]. Whether these guideline-directed cardiovascular treatment can also reduce systemic inflammatory or oxidative stress caused by chronic ulcer remains unknown and should be investigated in prospective studies.

The goal of emerging treatments must be to alter the inflammatory wound environment. Localized transdermal delivery systems that can deliver large molecular weight biologic drugs (such as nucleic acid and protein therapeutics) that can change gene expression in the cells located in the deep wound bed to change the pro-inflammatory microenvironment to an anti-inflammatory one are imperative for such an approach. Localized biologic drug delivery to the ulcer that focuses on altering macrophage polarization and cytokine signaling will reduce the duration of chronic inflammation and improve wound healing. Future studies should determine whether reducing wound inflammation also alters systemic inflammatory markers or cardiovascular outcomes.

Taken together, these considerations support an integrated model of care rather than a purely wound-centered approach. Patients with chronic ulcers can be considered for appropriate cardiovascular risk assessment and optimization of comorbidities, with further cardiac evaluation when clinically indicated. Likewise, patients with advanced CVD, frailty, immobility, diabetes, or peripheral vascular disease may benefit from proactive skin surveillance and early wound intervention. A multidisciplinary strategy involving wound specialists, vascular and cardiovascular clinicians, endocrinologists, rehabilitation teams, and infectious disease support when needed is likely to provide the greatest benefit. In this framework, chronic wound therapy should be viewed not only as local tissue management, but also as part of a broader effort to reduce systemic stress and improve overall outcomes.

## 7. Conclusions, Limitations and Conceptual Direction for the Field

The field is at a turning point. Although it is well recognized that cardiac dysfunction can impair wound healing, it remains unclear whether chronic wounds themselves may also exacerbate cardiac stress and dysfunction. Persistent inflammation, oxidative stress, infection-related immune activation, metabolic disturbance, maladaptive neurohumoral activation, and RAAS signaling collectively provide biological plausibility for an association between chronic wounds and cardiovascular stress. If validated, this would redefine chronic wounds as possible amplifiers of cardiovascular decline rather than merely peripheral consequences of systemic disease.

This review selectively narrates the heterogenous wound type, patient population, study design, and cardiovascular outcome. However, prospective, longitudinal, and interventional studies remain limited. In addition, shared comorbidities, including diabetes, obesity, vascular disease, renal dysfunction, frailty, infection, and immobility, make it difficult to distinguish the independent contribution of the wound to CVD from other risk factors of CVD. Several proposed mechanisms are also based on the integration of separate wound-healing and cardiovascular studies rather than on direct demonstration of wound-derived signals reaching or affecting the heart.

Collectively, the available evidence supports the biological plausibility of a wound–heart axis but does not yet establish that chronic wounds are independent risk factors for various pathologies that affect the heart and vasculature.

The next phase of research must move from association to causation. Key priorities include determining whether wound healing lowers cardiovascular risk, identifying the mediators of wound-to-heart signaling, defining the reversibility of cardiac changes, and testing targeted interventions that may improve both wound and cardiovascular outcomes. Together, these efforts could establish the wound–heart axis as an important translational and clinical paradigm.

## Figures and Tables

**Table 3:** Representative wound-associated miRNAs with documented roles in cardiovascular injury or remodeling.

miRNA	Pathological Context	Condition/Model	Association/Functional Interpretation	Key Reference
**miR-23a-3p**	Wound healing	Hypoxia-associated angiogenesis	Increases vascular permeability and angiogenesis by targeting prolyl hydroxylase and ZO-1 under hypoxia	Hsu et al. [72]
		Mouse burn wound model	Downregulated in injured skin vs. normal skin	Mu-Han et al. [73]
		Human DFU tissue (RNA-seq)	Differentially expressed in DFUs; not consistently up or down	Vikrant Rai [74]
	Cardiac damage	H9c2 cardiomyocytes (high glucose)	Protective: reduces apoptosis, enhances survival	Wu et al. [75]
		Ischemia/reperfusion injury (rat)	Upregulated in injured myocardium; inhibition reduces infarct and apoptosis	Cheng et al. [76]
		STEMI (human patients)	4.857-fold lower	Bukauskas et al. [77]
**miR-21-5p/miR-21-3p**	Wound healing	Skin wound (rat)	Upregulated; Promotes proinflammatory M1 macrophage polarization, dendritic cell differentiation, and fibroblasts production of IL-6 and IL-8; PTEN, RECK; Improves wound healing in aged mice and diabetic rats	Han et al. [78]
		miR-21 modulation in wound models (mouse)	Promotes re-epithelialization and granulation tissue formation	Wang et al. [79]
	Cardiac damage	Cardiac fibroblasts (heart failure model)	Upregulated; promotes fibrosis and maladaptive remodeling	Thum et al. [80]
		Cardiomyocytes ischemia/reperfusion (H9C2)	Protective in acute injury; reduces apoptosis via PTEN/Akt	Yang et al. [81]
		CHF-miR-21-3p specific (CHF patients, rat, H9C2)	Upregulated; promotes macrophage polarization, mitophagy, myocardial structural disruption and fibrosis	Huang et al. [82]
		Circulating miR-21 in Acute MI (human patients)	Elevated levels correlate with injury	Wang et al. [83]
**miR-126-3p**	Wound healing	Endothelial and diabetic wound contexts	Reduced in peripheral blood of diabetic patients with chronic non-healing ulcers vs. non-ulcer diabetic controls; associated with endothelial repair and angiogenesis	Rasmi et al. [84]
		EndMT model	Downregulated during endothelial injury/transition; overexpression mitigates EndMT and maintains endothelial phenotype (angiogenesis, migration)	Jordan et al. [85]
	Cardiac/cardiovascular pathology	Hypertensive general population (Hortega cohort)	Elevated plasma level associated with increased cardiovascular events, coronary artery disease and stroke risk; suggests potential prognostic biomarker	Martinez-Arroyo et al. [86]
		Heart failure risk in population cohort	Higher circulating miR-126-3p associated with increased heart failure risk; Mendelian randomization supports potential causal association	Grau-Perez et al. [87]
		AMI patients-EV/serum studies	Lower extracellular vesicle–associated miR-126 (including 3p) observed in AMI vs. stable CAD controls; changes in EV-miR-126 linked to severity/prognosis	Yuan et al. [88]
		Endothelial integrity and angiogenesis studies	Maintains vascular integrity/angiogenesis; knockdown impaired ischemia-induced angiogenesis in animal models	Wang et al. [89]
**miR-150 (miR-150-5p)**	Wound healing	MSCs-derived exosomal miR-150-5p in skin wounds	Promotes skin wound healing by activating the PI3K/AKT pathway through *PTEN* suppression- enhances keratinocyte and fibroblast functions and accelerates closure	Xiu et al. [90]
	Cardiac/cardiovascular pathology	Acute myocardial infarction-mouse and human	Reduced after AMI; overexpression improves cardiac function, decreases infarct size, inhibits apoptosis, and reduces Ly-6C(high) monocyte infiltration by targeting *CXCR4*	Liu et al. [91]
	Cardiac damage	Cardiomyocyte survival in ischemia	Protects against ischemia-induced injury by enhancing cardiomyocyte survival and reducing apoptosis	Tang et al. [92]
	Cardiac remodeling and fibrosis	Myofibroblast-specific miR-150 deletion (mouse MI)	Loss of miR-150 exacerbates cardiac dysfunction, apoptosis, and fibrosis after chronic MI; indicates a protective role	Kawaguchi et al. [93]
	Heart failure (general)	Human CVD and HF	Downregulated in patients with various cardiovascular diseases (HF, AMI, DCM, ischemic cardiomyopathy); lower levels correlate with disease severity and outcomes	Aonuma et al. [94]
**miR-93-3p**	Wound healing	BMSC derived exosomes + skin injury (HaCaT cells, H_2_O_2_ model)	Upregulated in BMSC-exo treatment promotes keratinocyte proliferation and migration and suppresses apoptosis via targeting *APAF1*; loss of miR-93-3p reduces those effects	Shen et al. [95]
	Wound healing (anal fistula surgery model)	Post-surgical wound + exosomes (miR-93-3p)	Upregulated in exosomal delivery promotes M2 macrophage polarization, reduces pro-inflammatory cytokines, accelerates healing; targets *EIF4EBP1*	Zhang et al. [96]
	Cardiac/cardiomyocyte injury	H9c2 cardiomyocytes + LPS	Overexpression protects against LPS-induced inflammation and apoptosis in cardiomyocytes by inhibiting the TLR4 pathway	Tang et al. [97]
	Inflammation-associated cardiac damage (sepsis)	Septic cardiomyopathy review context	Can inhibit TLR4 signaling and protect against inflammation/apoptosis in cardiomyocytes	Wu et al. [98]
miR-29 family (miR-29a/b/c)	Wound healing/fibrotic skin contexts	Thermal injury/scald wound (rat)	Reduced in wound tissue; treatment with miR-29b promotes wound healing and reduces scar formation via suppressing collagen deposition and TGF-β1/Smad/CTGF signaling	Guo et al. [99]
		General fibrotic contexts (review)	miR-29 family members are generally downregulated in fibrotic skin diseases, and downregulation is associated with increased ECM gene expression; indicates anti-fibrotic roles	Harmanci et al. [100]
	Cardiac pathogenicity-post-myocardial infarction	Myocardial infarction (mouse)	Reduced in post-MI infarct border zone, de-repression of collagen genes, contributes to cardiac fibrosis; overexpression reduces fibrosis	van Rooij et al. [101]
	Cardiac remodeling (pressure overload)	Transverse aortic constriction (TAC) model (mouse)	Genetic inhibition/knockout of family attenuates cardiac hypertrophy, fibrosis, and dysfunction; promotes remodeling	Sassi et al. [102]
	Cardiac ECM/fibroblast regulation	Fibroblast ECM gene targeting	Target collagen and other ECM genes; higher miR-29, lower collagen expression, strong anti-fibrotic mechanism	Kriegel et al. [103]
	Cardiovascular disease review	Atherosclerosis, fibrosis, remodeling	Implicated in atherosclerosis, myocardial fibrosis, and heart failure via regulation of ECM and stress pathways	Liu et al. [104]
miR-125b (primarily miR-125b-5p)	Wound healing	Exosomal transfer in skin/fibroblast models	Exosome-delivered miR-125b promotes myofibroblast differentiation and wound healing by inhibiting *Sirt7* and enhancing fibroblast migration/proliferation	Xia et al. [105]
		Hypoxic ucMSC-derived exosomes + skin injury	Hypoxic exosomal miR-125b increases endothelial survival, proliferation, migration, and accelerates cutaneous wound closure by targeting *TP53INP1*	Zhang et al. [106]
	Cardiac pathogenicity	Cardiac fibrosis and fibroblast-to-myofibroblast transition	Upregulated in fibrotic hearts; promotes cardiac fibroblast proliferation and activation, partly by inhibiting p53 and apelin, driving fibrogenesis	Nagpal et al. [107]
		Cardiomyocyte apoptosis/Heart failure model (TAC/MI)	Downregulated in failing hearts; overexpression reduces cardiomyocyte apoptosis and improves function via targeting *BAK1*	Zhang et al. [108]
		Acute myocardial infarction cardioprotection	Protective in acute ischemia/reperfusion; knockdown worsens post-MI cardiac structure, stress, inflammation, and fibrosis	Bayoumi et al. [109]
		ACS (clinical)	Lower miR-125b linked with better long-term survival in ACS with multivessel disease-hypothesis-generating; needs larger cohorts	Gager et al. 2022 [110]
miR-145 (miR-145-5p)	Wound healing	Hypertrophic skin scars (human/TGF-β1 fibroblast model)	Three-fold upregulated in hypertrophic scar tissue and TGF-β1-induced myofibroblasts; promotes α-SMA expression and myofibroblast activity; miR-145 inhibition reduces collagen, TGF-β1 production, contractility, and migration	Gras et al. [111]
	Cardiac pathogenicity	MI and cardiac fibroblast function	miR-145 expression decreases early post-injury and then recovers; promotes cardiac fibroblast to myofibroblast differentiation, aiding scar contraction and healing; miR-145 KO impairs scar contraction and wound healing post-MI	Song et al. [112]
		Cardiac fibroblast → myofibroblast differentiation (mechanistic)	Drives differentiation of cardiac fibroblasts into myofibroblasts and organizes α-SMA stress fibers contributing to wound contraction/scar maturation post-cardiac injury	Wang et al. [113]
		Cardiac apoptosis/mitochondrial stress	miR-145 can modulate mitochondrial apoptotic pathways in cardiomyocytes under oxidative stress (suggesting a protective effect against apoptosis)	Li et al. [114]
miR-181a	Wound healing/vascular injury	Diabetic wound healing mouse model	Upregulated and suppresses *Prox1*, impairing lymphangiogenesis and delaying wound healing	He et al. [115]
	Cardiac fibrosis	Rat MI model	Involved in myocardial fibrosis; in MI models, it influences TGF-β receptor III (suggesting fibrotic pathway modulation), though functional mechanisms are still being explored.	Chen et al. [116]
	Cardiac-cardiomyocyte apoptosis/HF	DOX-induced HF and H/R models	miR-181c protects cardiomyocytes against apoptosis and injury via activation of the PI3K/Akt pathway in hypoxia/reoxygenation and doxorubicin-induced injury models, indicating a potential cardioprotective role.	Li et al. [117]
miR-20a	Wound healing	Acute wound repair/keratinocytes (mouse and human)	Upregulated with wound repair (in acute wounds versus chronic ulcers); miR-20a (together with miR-19a/b) limits inflammation in keratinocytes by reducing TLR3-mediated NF-κB activation, which promotes wound closure and reduces prolonged inflammation. Lack of miR-20a contributes to sustained inflammation and delayed healing.	Li et al. [118]
	Cardiac-cardiomyocyte apoptosis/stress response	Primary cardiomyocytes (NRCM) stress/hypoxia model	Upregulated under acute biomechanical stress and hypoxia; inhibits hypoxia-induced cardiomyocyte apoptosis, likely by targeting *Egln3*/PHD3, indicating a cardioprotective role against remodeling and apoptosis.	Frank et al. [119]
	Cardiac development/differentiation (in vitro)	P19 embryonic cardiac differentiation model	Downregulated during cardiomyocyte differentiation; overexpression suppressed proliferation and differentiation and promoted apoptosis in P19 cells by targeting *SMO*, suggesting complex, stage-specific roles in cardiac development rather than adult injury repair.	Ai et al. [120]
miR-146a/miR-146a-5p	Wound healing-diabetic skin/ulcer models	Diabetic wounds in mice; KO/diabetic models	miR-146a deficiency delays wound healing; regulates inflammatory signaling, and its loss leads to prolonged inflammation and delayed skin wound closure.	Xu et al. [121]
		Diabetic ulcer macrophage polarization model	Overexpression promotes M2 macrophage polarization and enhances wound healing by inhibiting TLR4/NF-κB inflammatory axis in diabetic ulcers.	Peng et al. [122]
	Cardiac damage- inflammation and remodeling	Isoproterenol-induced cardiac fibrosis (rat)	miR-146a-5p is downregulated in ISO-induced cardiac fibrosis; overexpression attenuates fibrosis and reduces collagen and α-SMA via targeting *FGF2*.	Zhang et al. [123]
		Cardiac contractile dysfunction (sunitinib model)	Down regulation of miR-146a expression contributes to cardiac contractile dysfunction; upregulation alleviates dysfunction by targeting *PLN* and *ANK2* regulatory pathways.	Shen et al. [124]
		Circulating miR-146a levels-clinical UAP/CAD studies	Higher plasma miR-146a correlated with more severe coronary lesions and poorer prognosis in unstable angina and STEMI cohorts (biomarker evidence).	Shi et al. [125]
miR-199a-5p	Wound healing	Normal skin repair/angiogenesis	Downregulated during normal wound healing, which is necessary to derepress angiogenesis via the ETS-1–MMP1 pathway.	Chan et al. [126]
		Diabetic wound context (DFU animal or cell models)	Evidence suggests upregulation of miR-199a-5p in diabetic wound tissues and high-glucose models, where it inhibits proliferation/migration of endothelial cells and fibroblasts (e.g., via *VEGFA* and *ROCK1* inhibition), impairing normal wound healing.	Wang et al. [127]
	Cardiac damage-remodeling/fibrosis	Mouse *Angiotensin II* (Ang-II)-induced hypertrophy and fibrosis	Upregulated in hypertrophic and fibrotic heart tissue and contributes to cardiac remodeling by promoting hypertrophy and fibrosis through targets such as *Ppargc1a* and *Sirt1***.**	Zeng et al. [128]
	Cardiac damage-myocardial infarction/functional repair	Nanoparticle-mediated delivery in rodent MI model	Therapeutic delivery of miR-199a-5p after MI improved cardiac repair, reduced apoptosis, reduced infarct size, and improved contractile function, indicating a cardio-protective reparative effect in this context.	Cheng et al. [129]
	Cardiac injury-cardiomyocyte death/ferroptosis	Ischemia/hypoxia cardiomyocyte injury model	Promotes ferroptosis and cardiomyocyte death under ischemic/hypoxic conditions, demonstrating context-dependent pro-injury effects.	Zhang et al. [130]
miR-205-5p	Wound healing/scar formation	Diabetic foot ulcer (EV-carried)	Upregulated in diabetic foot ulcer wound fluid EVs; miR-205-5p inhibits angiogenesis by targeting *VEGFA*, thereby suppressing endothelial tube formation and wound angiogenesis.	Liu et al. [131]
	Cardiac injury/remodeling	Ischemia/reperfusion (MI/R) injury (mouse)	Increased miR-205 in injured hearts; inhibiting miR-205 reduces infarct size, improves cardiac function, and suppresses apoptosis, oxidative stress, and mitochondrial dysfunction.	Xu et al. [132]
		HFD-induced atrial fibrosis (mouse)	Decreased miR-205-5p levels associated with atrial fibrosis; upregulation reduces fibrosis, mitochondrial damage, and metabolic abnormalities via EHMT2/IGFBP3 axis.	Xiao et al. [133]
miR-135a	Wound healing	Exosomes from human amnion mesenchymal stem cells modulating wound repair	miR-135a promotes wound healing by increasing cell migration and wound closure, likely via downregulation of *LATS2*, which enhances migration required for repair.	Gao et al. [134]
	Cardiac damage- remodeling	ISO-induced cardiac injury (mouse)	miR-135a expression increases early then declines; overexpression protects against ISO-induced cardiac injury, fibrosis, inflammation, and apoptosis via TLR4 pathway inhibition.	Feng et al. [135]
	Cardiac injury-I/R- diabetic state	Myocardial ischemia/reperfusion injury in diabetic mice	Downregulated after I/R in diabetic hearts, and overexpression reduces infarct size, apoptosis, and oxidative stress via targeting *TXNIP*-indicating a protective role.	Zhu et al. [136]
	Cardiac remodeling/fibrosis	Iso-induced cardiac fibrosis via TRPM7–collagen pathway (in vitro)	Inhibits cardiac fibrosis in vitro by downregulating TRPM7-collagen I and related profibrotic signaling, suggesting anti-fibrotic effects in heart stress contexts.	Wu et al. [137]
miR-152-3p	Wound healing	DFU-human and mouse diabetic wounds	Upregulated in DFU patient blood/tissues; increases with high glucose and after wounding in diabetic mice; *miR-152-3p* Suppresses *PTEN*, impairing endothelial function (proliferation/angiogenesis) and delays wound healing. Antagonizing miR-152-3p improves endothelial proliferation, angiogenesis, and wound repair.	Xu et al. [138]
	Cardiac damage-ischemia-related	Ischemia/reperfusion injury (brain endothelial model)	Downregulated after ischemic injury (OGD/R) in endothelial cells (Bend.3); overexpression reduces apoptosis, preserves barrier integrity (TEER), and inhibits the JNK/c-Jun pro-apoptotic pathway *by* targeting *MAP3K2*, indicating a protective anti-apoptotic role in endothelial ischemic injury.	Li et al. [139]
miR-200a-3p	Wound healing	Diabetic patient wound	Downregulated in the wound, but the wound healing role is not well established	Li et al. [140]
miR-200c-3p	Wound healing- diabetic wounds/epithelial cells	Diabetic foot ulcers and keratinocytes (HaCaT), diabetic mouse model	Increased miR-200c; ROS-induced upregulation delays wound healing: inhibition of miR-200c (anti-miR) accelerates keratinocyte proliferation and closure; targets *SIRT1*, *FOXO1*, *ZEB1*, reduces oxidative stress and apoptosis; anti-miR prolongs healing.	DAgostino et al. [141]
miR-200a-3p	Cardiac injury-CME	Rat CME model	Downregulated after CME, and overexpression attenuates myocardial injury, improves contractility, reduces infarct size and cardiomyocyte apoptosis, suppresses oxidative stress and pyroptosis via TXNIP/NLRP3 inhibition.	Chen et al. [142]
	Cardiotoxicity- DOX model	Doxorubicin cardiotoxicity in rat	Increased miR-200a-3p contributes to cardiotoxicity; inhibition alleviates damage (reduces CK-MB/LDH, improves hemodynamics), associated with *PEG3* regulation in cell/animal models.	Fu et al. [143]
miR-200c-3p	Cardiac remodeling-chronic pressure overload/hypertrophy	Mice subjected to pressure overload	miR-200c-3p enriched in cardiomyocyte EVs, impairs endothelial function and worsens remodeling; antagomir against miR-200c-3p reduces hypertrophy, fibrosis, preserves capillary density and function.	Ottaviani et al. [144]
miR-200c	Cardiac ischemia/reperfusion (I/R)	Rat MI/R and H9c2 cardiomyocyte model	Increased after I/R and ROS induces miR-200c; overexpression worsens I/R injury via effects on glutamine metabolism and increases ROS/apoptosis.	Liu et al. [145]
miR-221-3p	Wound healing	Mouse excisional and diabetic wound models	Increased miR-221-3p promotes wound healing by suppressing inflammatory cytokines (IL-1β, IL-6, IL-8, TNF-α), reducing neutrophil/macrophage infiltration and shifting macrophage phenotype, and inhibiting high-glucose-induced inflammation in keratinocytes via targeting *DYRK1A*/STAT3 signaling.	Hu et al. [146]
	Cardiovascular-endothelial repair and EPC function	EPCs treated with LP(a) (in vitro)	Negatively regulates EPC function; inhibition restores proliferation, migration, adhesion, and angiogenesis, in part by upregulating *SIRT1* and activating RAF/MEK/ERK signaling- suggesting pro-angiogenic and repair-supportive effects when miR-221-3p is low/inhibited.	Zhang et al. [147]
	Cardiac and vascular disease	Review/multiple disease contexts	miR-221 family (including 3p) can affect angiogenesis by reducing endothelial NO and interacting in wound healing/fibrosis processes broadly, though detailed wound vs. cardiac functions require more model-specific data.	Chistiakov et al. [148]
miR-223-3p	Wound healing	Diabetic patient wound	Upregulated, but wound healing role is not well established.	Li et al. [140]
	Cardiac ischemia/myocarditis/apoptosis	MI models (rats; cardiomyocytes)	Downregulated in MI/injury, and its overexpression reduces inflammation and apoptosis, reducing myocarditis and improving cardiac function by targeting *FBXW7***.**	Zhang et al. [149]
	Cardiovascular disease and atherosclerosis	Atherosclerosis/plaque and lipid models	miR-223-3p regulates cholesterol metabolism, endothelial inflammation, macrophage activity, and vascular remodeling; circulating levels associate with atherosclerosis severity and may serve as biomarkers.	Zhang et al. [150]
	Cardiac hypertrophy and heart failure	Transgenic and hypertrophy models; CVD patients	miR-223-3p shows mixed regulation: can be downregulated in early hypertrophy models but is upregulated in advanced/diseased hearts and associated with cardiac hypertrophy, fibrosis, arrhythmia, and poor outcomes; may both promote and limit pathology depending on stimuli.	Zhang et al. [150]
miR-423-3p	Wound healing	Mouse full-thickness cutaneous wound model	Enriched in plasma-derived small extracellular vesicles, associated with reduced angiogenesis and delayed wound closure.	Fu-Xing et al. [151]
	Cardiovascular- CAD/population risk	General population cohort (serum miRNAs)	Circulating miR-423-3p improves prediction of primary CAD outcomes beyond traditional risk factors; higher baseline levels associated with incident CAD events over long-term follow-up.	Wang et al. [152]
	Cardiac tissue expression changes- CHD surgery	Atrial myocardial samples before vs. after corrective surgery in CHD	miR-423-3p (alongside other miRNAs) is downregulated following cardiopulmonary bypass in cardiac tissue of neonate/infant CHD patients, suggesting responsiveness to surgical and hemodynamic stress.	Abu-Halima et al. [153]
	Cardiac biomarker context (indirect)	General CAD/HF plasma profiling	miR-423 (precursor) and related strands (often 5p) have been investigated extensively as circulating biomarkers in heart failure and coronary disease, although many reports focus on miR-423-5p.	Rizzacasa et al. [154]
	Cardiovascular biomarker meta-context	Epidemiologic CVD risk panels	Included in panels with other miRNAs (e.g., miR-126, miR-210) showing expression changes associated with CAD and risk profiling, suggesting its biomarker potential in cardiovascular disease.	Vavassori et al. [155]
miR-122-5p	Wound healing	Acute wound expression profiling	miR-122-5p is one of the very few miRNAs differentially expressed during acute wound healing vs. normal skin, suggesting a role in cutaneous repair regulation.	Li et al. [156]
	Wound healing-diabetic ulcers (preclinical)	Diabetic foot ulcer model	Upregulated in DFUs; miR-122-5p delays wound repair by disrupting re-epithelialization and enhancing inflammation, indicating a maladaptive role in impaired healing. (Note: currently low citation count; seek future validation).	Yuan et al. [157]
	Cardiac arrest/survival prognostic	Out-of-hospital cardiac arrest (clinical cohort)	Lower circulating levels of miR-122-5p at ~48 h post-arrest are independently associated with worse neurological outcome and survival, indicating prognostic biomarker value after severe cardiac injury.	Devaux et al. [158]
	Cardiovascular disease- TAVR and LVEF	Transcatheter aortic valve replacement patients	Higher miR-122-5p levels associate with reduced left ventricular function (EF) and increased cardiovascular mortality over long-term follow-up, suggesting a negative correlation with cardiac function in structural heart disease.	Hosen et al. [159]
	Cardiac injury biomarkers (polytrauma)	Polytrauma with myocardial injury	miR-122-5p is upregulated in plasma of patients with elevated troponin and right ventricular dysfunction post-polytrauma, indicating potential as an early biomarker of myocardial injury/inflammation.	Han et al. [160]
	Cardiac inflammation and damage	Sepsis-triggered myocardial injury (preclinical)	Inhibition of miR-122-5p mitigates inflammatory injury, oxidative stress, and apoptosis in models of sepsis-induced myocardial injury (targeting *GIT1*), suggesting functional pathogenic involvement.	Song et al. [161]
	Cardiovascular fibrosis and remodeling (review)	Review of cardiovascular disease models	miR-122 (including 5p strand) is implicated in cardiovascular fibrosis, pathological remodeling, inflammation, apoptosis, and dysfunction across HF, atherosclerosis, MI, and hypertension, often promoting maladaptive processes.	Liu et al. [162]

Note: miRNA: MicroRNA; ZO-1: Zonula occludens-1; DFU: Diabetic foot ulcer; RNA-seq: Ribonucleic acid sequencing; STEMI: ST-elevation myocardial infarction; M1: Classically activated/pro-inflammatory macrophage phenotype; IL: Interleukin; PTEN: Phosphatase and tensin homolog; RECK: Reversion-inducing cysteine-rich protein with Kazal motifs; M2: Alternatively activated/pro-repair macrophage phenotype; CHF: Congestive heart failure; MI: Myocardial infarction; EndMT: Endothelial-to-mesenchymal transition; AMI: Acute myocardial infarction; EV: Extracellular vesicle; CAD: Coronary artery disease; MSC: Mesenchymal stem cell; PI3K: Phosphoinositide 3-kinase; AKT: Protein kinase B; *CXCR4*: C-X-C motif chemokine receptor 4; CVD: Cardiovascular disease; HF: Heart failure; DCM: Dilated cardiomyopathy; BMSC: Bone marrow mesenchymal stem cell; H_2_O_2_: Hydrogen peroxide; *APAF1*: Apoptotic peptidase activating factor 1; *EIF4EBP1*: Eukaryotic translation initiation factor 4E-binding protein 1; LPS: Lipopolysaccharide; TLR4: Toll-like receptor 4; TGF-β1: Transforming growth factor beta 1; CTGF: Connective tissue growth factor; ECM: Extracellular matrix; TAC: Transverse aortic constriction; Sirt7: Sirtuin 7; ucMSC: Umbilical cord mesenchymal stem cell; TP53INP1: Tumor protein p53 inducible nuclear protein 1; *BAK1*: BCL2 antagonist/killer 1; ACS: Acute coronary syndrome; α-SMA: Alpha-smooth muscle actin; KO: Knockout; *Prox1*: Prospero homeobox 1; DOX: Doxorubicin; H/R: Hypoxia/reoxygenation; TLR3: Toll-like receptor 3; NF-κB: Nuclear factor kappa B; NRCM: Neonatal rat cardiomyocyte; PHD3: Prolyl hydroxylase domain-containing protein 3; SMO: Smoothened; ISO: Isoproterenol; *FGF2*: Fibroblast growth factor 2; *PLN*: Phospholamban; *ANK2*: Ankyrin 2; UAP: Unstable angina pectoris; ETS-1: ETS proto-oncogene 1, transcription factor; MMP1: Matrix metalloproteinase 1; VEGFA: Vascular endothelial growth factor A; ROCK1: Rho-associated coiled-coil containing protein kinase 1; Ang-II: Angiotensin II; Ppargc1a: Peroxisome proliferator-activated receptor gamma coactivator 1-alpha; *Sirt1/SIRT1*: Sirtuin 1; MI/R: Myocardial ischemia/reperfusion; HFD: High-fat diet; EHMT2: Euchromatic histone lysine methyltransferase 2; IGFBP3: Insulin-like growth factor-binding protein 3; *LATS2*: Large tumor suppressor kinase 2; I/R: Ischemia/reperfusion; *TXNIP*: Thioredoxin-interacting protein; TRPM7: Transient receptor potential melastatin 7; OGD/R: Oxygen-glucose deprivation/reoxygenation; TEER: Transendothelial electrical resistance; JNK: c-Jun N-terminal kinase; MAP3K2: Mitogen-activated protein kinase kinase kinase 2; ROS: Reactive oxygen species; *FOXO1*: Forkhead box O1; *ZEB1*: Zinc finger E-box-binding homeobox 1; CME: Coronary microembolization; NLRP3: NLR family pyrin domain containing 3; CK-MB: Creatine kinase-MB; LDH: Lactate dehydrogenase; PEG3: Paternally expressed gene 3; IL-1β: Interleukin-1 beta; TNF-α: Tumor necrosis factor alpha; *DYRK1A*: Dual-specificity tyrosine-phosphorylation-regulated kinase 1A; STAT3: Signal transducer and activator of transcription 3; EPC: Endothelial progenitor cell; LP(a): Lipoprotein(a); RAF/MEK/ERK: RAF kinase/mitogen-activated protein kinase kinase/extracellular signal-regulated kinase signaling pathway; NO: Nitric oxide; *FBXW7*: F-box and WD repeat domain-containing 7; CHD: Congenital heart disease; TAVR: Transcatheter aortic valve replacement; LVEF: Left ventricular ejection fraction; EF: Ejection fraction; GIT1: G protein-coupled receptor kinase-interacting protein 1.

**Table 4:** Proteins identified in wound-derived extracellular vesicles (EVs) from diabetic foot ulcer and venous leg ulcer wound-dressing eluates and their known cardiovascular implications.

Protein	Known Function	Known Cardiovascular Implications	Supporting Literature
JUP	Desmosomal linker protein connecting cadherins to the cytoskeleton	Associated with arrhythmogenic cardiomyopathy and myocardial coupling defects	Swope et al. [163]; Li et al. [164]
DSP	Desmosomal structural protein	Linked to arrhythmogenic and dilated cardiomyopathy	Smith et al. [165]; Gasperetti et al. [166]
GSN	Cytoskeletal remodeling and inflammatory regulation	Altered during ischemia and heart failure	Patel et al. [167]
UBB	Protein degradation and quality control	Involved in cardiac stress responses	Pagan et al. [168]
C4A	Complement pathway protein	Contributes to ischemia/reperfusion inflammation	Vogel [169]
MPO	Oxidative enzyme from neutrophils	Strongly associated with atherosclerosis and adverse post-MI remodeling	Teng et al. [170]; Tang et al. [171]; Ramachandra et al. [172]; Mocatta et al. [173]
MMP9	ECM degradation and inflammatory remodeling	Contributes to post MI remodeling, adverse ventricular dilation, and fibrosis; MMP9 inhibition reduces infarct expansion	Ducharme et al. [174]; Lindsey et al. [175]; Frangogiannis [176]
MMP2	ECM remodeling protease	Major contributor to ischemia/reperfusion injury and cardiac remodeling; Promotes adverse ventricular remodeling	Wang et al. [177]; Cheung et al. [178]; Spinale [179]; Wolosowicz et al. [180]
MMP1	Collagen degradation enzyme	Associated with heart failure progression and plaque instability	King et al. [181]; Kelly et al. [182]
ANXA5	Membrane repair and apoptosis regulation	Protective associations in ischemia/reperfusion injury and cardiomyocyte survival; may inhibit apoptosis and inflammation after MI.	Jong et al. [183]; Zhao et al. [184]
S100A2	Calcium-binding protein involved in intracellular Ca^2+^ signaling and regulation of cellular functions	Experimental cardiac expression enhances Ca^2+^ cycling, cardiomyocyte contraction, and relaxation and partially restores contractile function in failing cardiomyocytes.	Wang et al. [185]
SERPINA1	Anti-inflammatory protease inhibitor	Protective against myocardial inflammation and ECM degradation	Toldo et al. [186]; Ambrosino et al. [187]
CD63	Exosome-associated tetraspanin	Modulates EV-mediated cardiac signaling	Thery et al. [188]
COL3A1	ECM fibrillar collagen	Involved in post-MI remodeling and vascular integrity	Pepin et al. [189]; Jugdutt et al. [190]

Note: JUP: Junction plakoglobin; DSP: Desmoplakin; GSN: Gelsolin; UBB: Ubiquitin B; C4A: Complement C4A; MPO: Myeloperoxidase; MMP: Matrix Metalloproteinase; ANXA5: Annexin A5; S100A2: S100 calcium binding protein A2; SERPINA1: Alpha 1 antitrypsin; CD63: Tetraspanin 30; COL3A1: Collagen α 1(III) chain.

## Data Availability

No new data were created or analyzed in this study. Data sharing is not applicable to this article.

## Abbreviations

The following abbreviations are used in this manuscript:

DFU	Diabetic foot ulcer
PU	Pressure ulcer
VLU	Venous leg ulcer
EV	Extracellular vesicle
CVD	Cardiovascular disease
ICU	Intensive care unit
ECG	Electrocardiogram
COPD	Chronic obstructive pulmonary disease
DVT	Deep vein thrombosis
CEAP	Clinical–etiological–anatomical–pathophysiological classification
PAD	Peripheral artery disease
MACE	Major adverse cardiovascular events
ANCA	Anti-neutrophil cytoplasmic antibody
RAAS	Renin–angiotensin–aldosterone system
MINS	Myocardial injury after non-cardiac surgery
TNF-α	Tumor necrosis factor-alpha
IL-1β	Interleukin-1 beta
IL-6	Interleukin-6
CRP	C-reactive protein
SERCA2a	Sarcoplasmic reticulum Ca^2+^-ATPase 2a
JAK	Janus kinase
STAT3	Signal transducer and activator of transcription 3
HFpEF	Heart failure with preserved ejection fraction
TGF-β	Transforming growth factor-beta
ROS	Reactive oxygen species
ATP	Adenosine triphosphate
DNA	Deoxyribonucleic acid
TLR4	Toll-like receptor 4
miRNA	MicroRNA
MMP	Matrix metalloproteinase
MPO	Myeloperoxidase
NT-proBNP	N-terminal pro-B-type natriuretic peptide
NF-κB	Nuclear factor kappa-B
AGE-RAGE	Advanced glycation end product-receptor for advanced glycation end products
MRI	Magnetic resonance imaging
ACE	Angiotensin-converting enzyme
